# Elevation of preoperative serum hs-CRP is an independent risk factor for malnutrition in patients with gastric cancer

**DOI:** 10.3389/fonc.2023.1173532

**Published:** 2023-05-24

**Authors:** Yuantian Mao, Jinlu Liu, Jiaming Li, Yue Qiu, Zhen Wang, Bopei Li, Siyu Liu, Lei Tian, Junqiang Chen

**Affiliations:** ^1^ Department of Gastrointestinal Surgery, The First Affiliated Hospital of Guangxi Medical University, Nanning, Guangxi Zhuang Autonomous Region, China; ^2^ Guangxi Key Laboratory of Enhanced Recovery After Surgery for Gastrointestinal Cancer, Nanning, Guangxi Zhuang Autonomous Region, China; ^3^ Guangxi Clinical Research Center for Enhanced Recovery After Surgery, Nanning, Guangxi Zhuang Autonomous Region, China; ^4^ Department of General Surgery, The Wuzhou Red Cross Hospital, Wuzhou, Guangxi Zhuang Autonomous Region, China

**Keywords:** Hs-CRP, gastric cancer, malnutrition, NRS2002, PG-SGA

## Abstract

**Background:**

Recent studies have reported hypersensitive C-reactive protein (hs-CRP) linked to clinicopathological characteristics and nutritional status of the tumor, but its clinical significance in GC remains unclear. This study aimed to investigate the relationship between preoperative serum hs-CRP level and clinicopathological features and nutritional status in gastric cancer (GC) patients.

**Methods:**

The clinical data of 628 GC patients who met the study criteria were analyzed retrospectively. The preoperative serum hs-CRP level was divided into two groups (<1 mg/L and ≥1 mg/L) to evaluate clinical indicators. Nutritional Risk Screening and nutritional assessment of GC patients were performed by the Nutritional Risk Screening 2002 (NRS2002) and the Patient-Generated Subjective Global Assessment (PG-SGA), respectively. The data were subjected to chi-square test, univariate and multivariate logistic regression analyses, respectively.

**Results:**

The analysis of 628 GC cases revealed that 338 patients (53.8%) were on malnutrition risk(NRS2002≥3 points), and 526(83.8%) had suspected/moderate to severe malnutrition(PG-SGA≥ 2 points). Preoperative serum hs-CRP level was significantly correlated with age, tumor maximum diameter (TMD), peripheral nerve invasion (PNI), lymph-vascular invasion (LVI), depth of tumor invasion (DTI), lymph node metastasis (LNM), pTNM stage, body weight loss (BWL), body mass index (BMI), NRS2002 score, PG-SGA grade, hemoglobin (HB), total protein (TP), albumin (ALB), prealbumin (PAB) and total lymphocyte count (TLC). Multivariate logistic regression analysis revealed that hs-CRP (OR=1.814, 95%CI=1.174-2.803; *P*=0.007), age, ALB, BMI, BWL and TMD were independent risk factors for existing malnutritional risk in GC. Similarly, non-malnutrition and suspected/moderate to severe malnutrition groups presented that hs-CRP (OR=3.346, 95%CI=1.833-6.122; *P*< 0.001), age, HB, ALB, BMI and BWL were independent risk factors for malnutrition in GC.

**Conclusion:**

In addition to the generally used nutritional evaluation indicators such as age, ALB, BMI, and BWL, the hs-CRP level may be used as a nutritional screening and evaluation indicator for GC patients.

## Introduction

Gastric cancer (GC) is regarded as one of the most common types of cancer universally. As per global cancer statistics, approximately one million new cases of GC were recorded in 2020 only, with an estimated 769,000 deaths, making GC the 5^th^ ranked cancer globally and 4^th^ for global mortality. The prevalence rates were highest in Eastern Asia and Eastern Europe, and rates in Northern America and Northern Europe were generally low and comparable to those reported in the African regions (1). Currently, the overall five-year survival rate for GC is less than 40%, with several predisposing factors such as tumor size, location, depth of invasion, lymph node metastasis, and distant metastasis (2–4). The nutritional status of patients also has a substantial impact on the therapeutic effect of tumor. GC patients had varying degrees of food intake disorders due to nausea, vomiting, abdominal pain and gastrointestinal obstruction, and the incidence of this malnutrition could be as high as 60% (5). Malnutrition has been linked to an increased risk of postoperative complications in GC patients and a poor prognosis (6, 7). Wischmeyer et al. (8) conducted a survey and discovered that 2/3 of patients who underwent gastrointestinal surgery were malnourished, complications and mortality in malnourished patients increased by three to five times. Nutritional treatment for inpatients could have saved 52 dollars in hospital costs. Still, only 1/5 of hospitals carried out regular nutritional screening, and only 1/5 of patients received nutritional treatment before surgery. Therefore, preoperative nutritional screening and evaluation of GC patients are crucial for timely nutritional intervention. There are numerous indicators and tools available for clinical nutrition screening and evaluation today, with Nutritional Risk Screening 2002 (NRS2002) being a popular and effective nutrition screening tool (9, 10). Patient-Generated Subjective Global Assessment (PG-SGA) is a commonly used tool recommended by the American Society of Parenteral and Enteral Nutrition (ASPEN) for estimating tumor nutrition and can effectively assess the nutritional status of tumor patients (11, 12). However, it was discovered that these nutritional indicators and assessment tools have some limitations in the clinical work, and more nutrition-related indicators are required to reflect the nutritional status of tumor patients more comprehensively.

C-reactive protein (CRP) is a non-specific acute stage protein produced by the liver, which plays an important role in the inflammatory response and can be used as a biological marker of inflammation (13). However, due to limitations in detecting the sensitivity of standard CRP, low serum CRP levels are difficult to detect, potentially overlooking the clinical value of undetected low levels of CRP in tumors. The hypersensitive C-reactive protein (hs-CRP) is more sensitive than the standard CRP detection, which can detect the low level of CRP in serum. Therefore, it objectively reflected the serum level of CRP with superior clinical application value (14). Previous research focused solely on the role of hs-CRP in inflammation, but it has been found that hs-CRP is more than just an inflammatory indicator (15). It has been associated with numerous diseases in recent years, such as coronary heart disease (16), intracranial arterial stenosis (17), metabolic syndrome (18), and tumor (13, 19). In a study of GC, it was discovered that preoperative and postoperative CRP were related to poor prognosis (20). However, scarce data demonstrated the relationship between preoperative hs-CRP, nutritional status, and clinicopathological characteristics of GC patients, which should be further investigated. This study explored the connection between hs-CRP and GC from the aforementioned aspects and found that hs-CRP had a good clinical application value in the progression of GC and nutritional assessment.

## Methods

### Study design

This study protocol was approved by the ethics committee at the First Affiliated Hospital of Guangxi Medical University, and written informed consent was obtained from all the participants. The clinical data of 628 GC patients who met the inclusion criteria in the First Affiliated Hospital of Guangxi Medical University from December 2015 to February 2022 were collected. The preoperative serum hs-CRP level was divided into two groups (< 1 mg/L and ≥ 1 mg/L) to evaluate clinical indicators. Inclusion criteria included the followings: age ≥ 18 years, clear mind and the ability to appropriately answer questions, GC diagnosed by pathology, patients who underwent radical gastrectomy or palliative gastrectomy, and patients with complete study data. Exclusion criteria were as follows: patients who could not correctly describe their history, emergency surgical patients, patients with rheumatic immune diseases, hyperthyroidism, hepatitis, and other infectious diseases, patients with distant metastasis or with other malignancies.

NRS2002 score method was used for the nutritional risk screening, including three aspects: disease, nutritional status, and age, and a score ≥ 3 points indicated the risk of malnutrition (10). According to NRS2002, the patients were divided into two groups: the non-malnutrition risk group (< 3 points) and the existing malnutrition risk group (≥ 3 points). PG-SGA score was taken for the nutritional assessment, consisting of seven aspects: body weight, eating status, symptoms, activity and physical function, disease and nutritional needs, metabolic needs, and physical examination (21). Meanwhile, the comprehensive evaluation of PG-SGA was compromised of qualitative and quantitative evaluations. The relationship between qualitative evaluation and quantitative evaluation was as follows: 0-1 points represented good nutrition (grade A), 2-8 points represented suspected or moderate malnutrition (grade B), and more than 9 points represented severe malnutrition (grade C). As per the PG-SGA, they were also put into two groups: non-malnutrition group (grade A, 0-1 points) and suspected/moderate and severe malnutrition group (grade B + C, ≥ 2 points). All above scores were evaluated by doctors and nurses who were properly trained as per defined standards.

### Data collection

The data were collected from patients three days before surgery. The age, sex, hs-CRP, total lymphocyte count (TLC), hemoglobin (HB), total protein (TP), albumin (ALB), prealbumin (PAB), body weight loss (BWL), body mass index (BMI), NRS2002 score, and PG-SGA score were recorded. BMI was reported as current weight (kg)/height (m)^2^, BWL was calculated as (current weight - original weight)/original weight×100%, tumor patients who lost more than 2.4% of their involuntary weight within six months were considered to have weight loss (22).

Fasting blood samples for preoperative biochemical tests were taken from the patients and sent to the laboratory for examination on the morning of the second day after admission. The hs-CRP, TLC, and HB were detected by Backman Coulter LH780 automatic blood cell analyzer, and TP, ALB, and PAB were assessed by Hitachi 7600 automatic biochemical analyzer. The chief physician or associate chief physician of pathologists reported postoperative pathological conclusions.

The postoperative pathological data collected from the patients included the following: tumor maximum diameter (TMD), depth of tumor invasion (DTI), lymph node metastasis (LNM), peripheral nerve invasion (PNI), and lymph-vascular invasion (LVI), tumor grade, and tumor pathological TNM (pTNM) stage. GC staging was based on the eighth edition pTNM staging system jointly promulgated by the Union for International Cancer Control (UICC) and the American Joint Committee on Cancer (AJCC).

### Statistical analysis

SPSS 26.0 statistical software was used for data analysis. The measurement data were expressed as median and quartiles, and a non-parametric rank-sum test was utilized to check the differences in measurement data between the two groups. Chi-squared test (χ^2^ test) was done to compare the variances of enumeration data between the two groups. Univariate and multivariate analyses were performed by logistic regression. The results were considered significant if *P* < 0.05.

## Results

### Preoperative serum hs-CRP was associated with clinicopathological features of GC

The results included 628 GC cases. Among which, 400 (63.7%) were male subjects. Among total GC cases, 153 (24.4%) were well-differentiated or moderately differentiated cases, while 475 (75.6%) were poorly differentiated or undifferentiated cases. A total of 204 (32.5%) patients were having TMD ≥ 5 cm, 251 (40.0%) patients with PNI, 244 (38.9%) LVI, 146 (23.2%) T_0-1_ patients with DTI, 96 (15.3%) T_2_, 183 (29.1%) T_3_, and 203 (32.4%) T_4_. LNM was found in 214 (34.1%) cases with N0, 109 (17.4%) with N1, 113 (18.0%) with N2 and 192 (30.5%) with N3. According to pTNM stage, there were 311 (49.5%) patients at stage 0/I/II, 317 (50.5%) at stage III/IV. By nonparametric rank sum test and Chi-square analysis, the results showed that preoperative serum hs-CRP level was significantly correlated with age (*P* < 0.001), TMD (*P* < 0.001), PNI (*P* = 0.004), LVI (*P* = 0.001), DTI (*P* < 0.001), LNM (*P* = 0.001) and pTNM stage (*P* < 0.001, while there was no significant correlation between gender and tumor grade, as shown in **Table 1**.

**Table 1:** Relationship between preoperative serum hs-CRP and clinicopathological characteristics of GC patients.

Variables	Aggregate (n=628)	hs-CRP<1mg/L (n=299)	hs-CRP≥1mg/L (n=329)	χ^2^/U Value	*P* Value
Age(y)	–	52.0 (45.0,60.0)	60.0 (51.0,67.0)	33363.0	<0.001
Gende r(n)					
Male	400	180	220	3.012	0.083
Female	228	119	109		
Grade (n)					
Well/moderate	153	72	81	0.025	0.875
Poor/undifferentiated	475	227	248		
TMD(n)					
<5cm	424	230	194	23.029	<0.001
≥5cm	204	69	135		
PNI(n)					
No	377	197	180	8.153	0.004
Yes	251	102	149		
LVI(n)					
No	384	204	180	12.045	0.001
Yes	244	95	149		
DTI(n)					
T_0_/T_1_	146	95	51	29.893	<0.001
T_2_	96	49	47		
T_3_	183	65	118		
T_4_	203	90	113		
LNM(n)					
N_0_	214	125	89	15.487	0.001
N_1_	109	47	62		
N_2_	113	45	68		
N_3_	192	82	110		
pTNM stages(n)					
0/I/II	311	172	139	14.622	<0.001
III/IV	317	127	190		

hs-CRP, hypersensitive C-reactive protein; TMD, tumor maximum diameter; PNI, peripheral nerve invasion; LVI, lymph-vascular invasion; DTI, depth of tumor invasion; LNM, lymph node metastasis.

### Preoperative serum hs-CRP was related to the nutritional status of GC

Of 628 GC cases, 203 (32.3%) had BWL > 2.4%, 76 (12.1%) cases with BMI < 18.5 kg/m^2^, 402 (64.0%) cases with 18.5 kg/m^2^ ≤ BMI < 23.9 kg/m^2^, 150 (23.9%) cases with BMI ≥ 24 kg/m^2^, 338 (53.8%) cases with NRS2002 score ≥ 3 points, 526 (83.8%) cases were rated B/C grades in PG-SGA nutritional assessment. Chi-square test indicated that preoperative serum hs-CRP was significantly associated with BWL (*P* = 0.012), BMI (*P* < 0.001), NRS2002 score (*P* < 0.001) and PG-SGA nutritional grade (*P* < 0.001), while the nonparametric rank sum test analysis displayed that preoperative serum hs-CRP was significantly interrelated with HB (*P* < 0.001), TP (*P* = 0.001), ALB (*P* < 0.001), PAB (*P* < 0.001) and TLC (*P* = 0.001) in GC patients, as indicated in **Table 2**.

**Table 2:** Relationship between preoperative serum hs-CRP and some nutrition-related indicators of GC patients .

Variables	Aggregate (n=628)	hs-CRP<1mg/L (n=299)	hs-CRP≥1mg/L (n=329)	χ2/U Value	*P* Value
BWL(n)					
≤2.4%	425	217	208	6.264	0.012
>2.4%	203	82	121		
BMI (kg/m2)(n)					
<18.5	76	26	50	29.504	<0.001
18.5-23.9	402	224	178		
≥24	150	49	101		
NRS2002 scores(n)					
<3 points	290	166	124	20.033	<0.001
≥3 points	338	133	205		
PG-SGA grades(n)					
A	102	74	28	30.362	<0.001
B+C	526	225	301		
HB (g/L)	–	126.0 (110.9,136.6)	116.3 (92.1,130.1)	37725.5	<0.001
ALB (g/L)	–	38.8 (36.3,41.1)	36.4 (33.1,39.5)	32626.0	<0.001
TP (g/L)	–	64.2 (60.3,67.3)	62.3 (58.8,66.2)	41745.5	0.001
PAB (mg/L)	–	221.1 (193.1,269.0)	192.3 (158.5,230.7)	32376.5	<0.001
TLC (109/L)	–	1.82 (1.53,2.33)	1.69 (1.33,2.19)	41355.5	0.001

hs-CRP, hypersensitive C-reactive protein; BWL, body weight loss; BMI, body mass index; NRS2002,Nutritional Risk Screening 2002; PG-SGA,Patient-Generated Subjective Global Assessment; HB, hemoglobin; ALB, albumin; TP, total protein;PAB, prealbumin; TLC, total lymphocyte count.

### Preoperative serum hs-CRP used as an indicator of nutritional risk screening in GC patients

**Figure 1:**
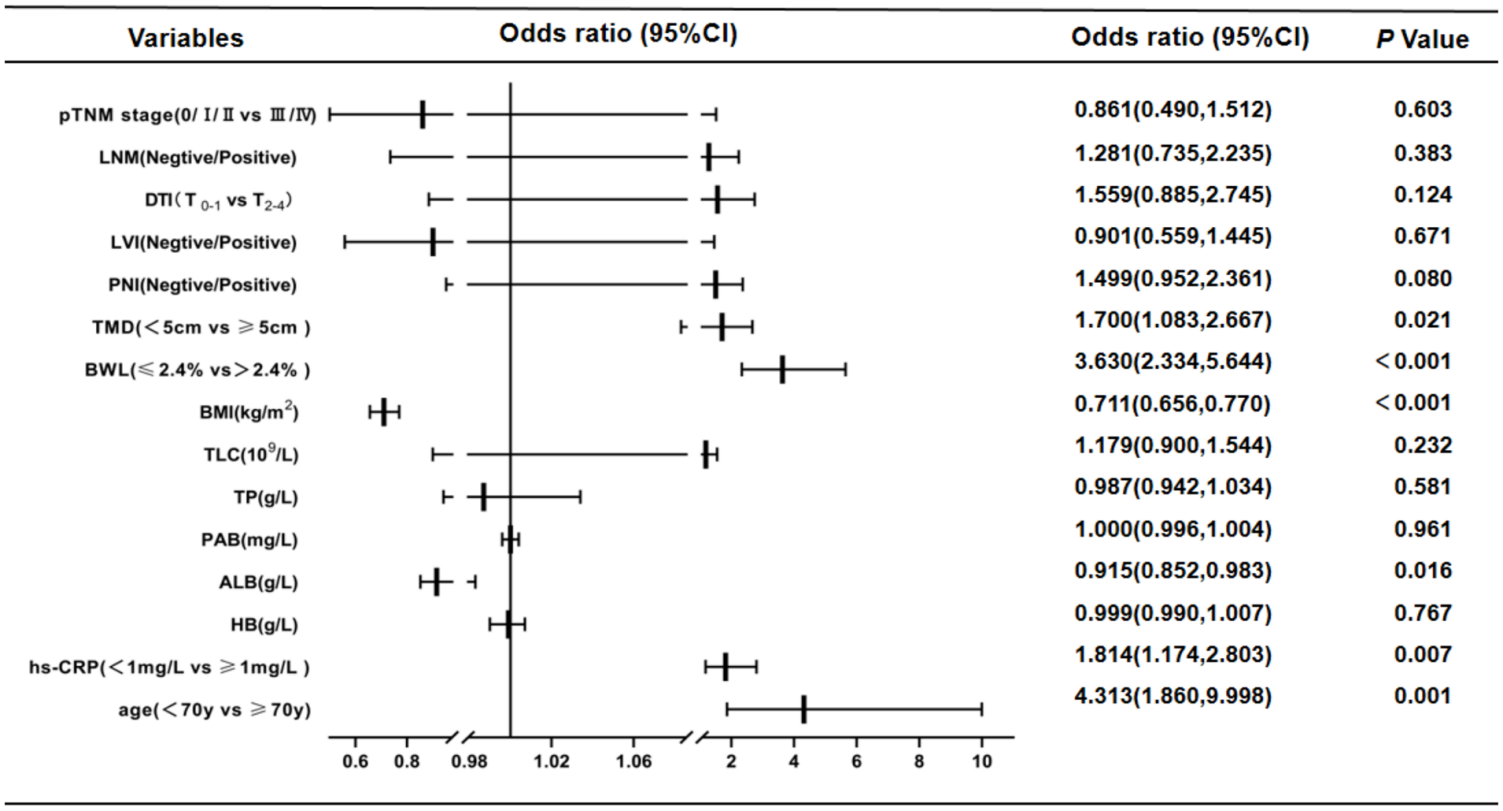
Results of multivariate Logistic regression analysis affecting nutritional risk in patients with GC by NRS2002 (scores<3 points vs. ≥ 3 points).This study showed that age, hs-CRP, ALB, BMI, BWL and TMD were independent risk factors for existing malnutrition risk in GC patients. CI, confidence interval; LNM, lymph node metastasis; DTI, depth of tumor invasion; LVI, lymph-vascular invasion; PNI, peripheral nerve invasion; TMD, tumor maximum diameter; BWL, body weight loss; BMI, body mass index; TLC, total lymphocyte count; TP, total protein; PAB, prealbumin; ALB, albumin; HB, hemoglobin; hs-CRP, hypersensitive C-reactive protein.

According to NRS2002 in this study, GC patients were divided into two groups: non-malnutrition risk (scores < 3 points) and existing malnutrition risk (scores ≥ 3 points). First, univariate logistic regression analysis were carried out, and the results were as follows: age (*P* < 0.001), hs-CRP (*P* < 0.001), HB (*P* < 0.001), ALB (*P* < 0.001), PAB (*P* < 0.001), TP (*P* < 0.001), TLC (*P* = 0.001), BMI (*P* < 0.001), BWL (*P* < 0.001), TMD (*P* < 0.001), PNI (*P* < 0.001), LVI (*P* < 0.001), DTI (*P* < 0.001), LNM (*P* = 0.001) and pTNM stages (*P* < 0.001). The aforementioned details were the risk factors of existing malnutrition risk in GC patients (**Table 3**). Then, multivariate logistic regression analysis was performed on the indicators of significant statistical differences obtained in the above from univariate analysis, and the results exhibited that age (OR= 4.313, 95% CI= 1.860 - 9.998; *P* = 0.001), hs-CRP (OR = 1.814, 95% CI = 1.174 - 2.803; *P* = 0.007), ALB (OR = 0.915, 95% CI = 0.852 - 0.983 P = 0.016), BMI (OR = 0.711, 95% CI = 0.656 - 0.770; *P* < 0.001), BWL (OR = 3.630, 95% CI = 2.334 - 5.644; *P* < 0.001) and TMD (OR = 1.700, 95% CI = 1.083 - 2.667; *P* = 0.021) were independent risk factors for existing malnutrition risk in GC patients (**Figure 1**).

**Table 3:** Univariate logistic regression analysis of nutritional risk in GC patients by NRS2002 (scores<3 points vs. ≥ 3 points).

Variables	Odds ratios (95% CI)	*P* Value
Gender	1.065 (0.769,1.477)	0.704
Age(<70 y vs. ≥70y)	4.823 (2.470,9.416)	<0.001
hs-CRP(<1.00mg/L vs. ≥1.00mg/L)	2.063 (1.500,2.839)	<0.001
HB	0.984 (0.978,0.991)	<0.001
ALB	0.852 (0.817,0.890)	<0.001
PAB	0.991 (0.988,0.994)	<0.001
TP	0.917 (0.890,0.944)	<0.001
TLC	0.686 (0.550,0.856)	0.001
BMI	0.738 (0.691,0.787)	<0.001
BWL(≤2.4%/>2.4%)	4.432 (3.035,6.471)	<0.001
Grade(Well/moderate vs. Poor/undifferentiated)	1.290 (0.896,1.858)	0.171
TMD(<5cm/≥5cm)	2.026 (1.435,2.861)	<0.001
PNI(Negtive/Positive)	1.920 (1.389,2.655)	<0.001
LVI(Negtive/Positive)	1.809 (1.303,2.510)	<0.001
DTI(T_0-1_ vs. T_2-4_)	2.850 (1.935,4.198)	<0.001
LNM(Negtive/Positive)	1.729 (1.240,2.412)	0.001
pTNM stages (0/I/II vs. III/IV)	1.782 (1.298,2.447)	<0.001

CI, confidence interval; hs-CRP, hypersensitive C-reactive protein; HB, hemoglobin; ALB, albumin; PAB, prealbumin; TP, total protein; TLC, total lymphocyte count; BMI, body mass index; BWL, body weight loss; TMD, tumor maximum diameter; PNI, peripheral nerve invasion; LVI, lymph-vascular invasion; DTI, depth of tumor invasion; LNM, lymph node metastasis.

### Preoperative serum hs-CRP used as a nutritional evaluation factor in GC patients

**Figure 2:**
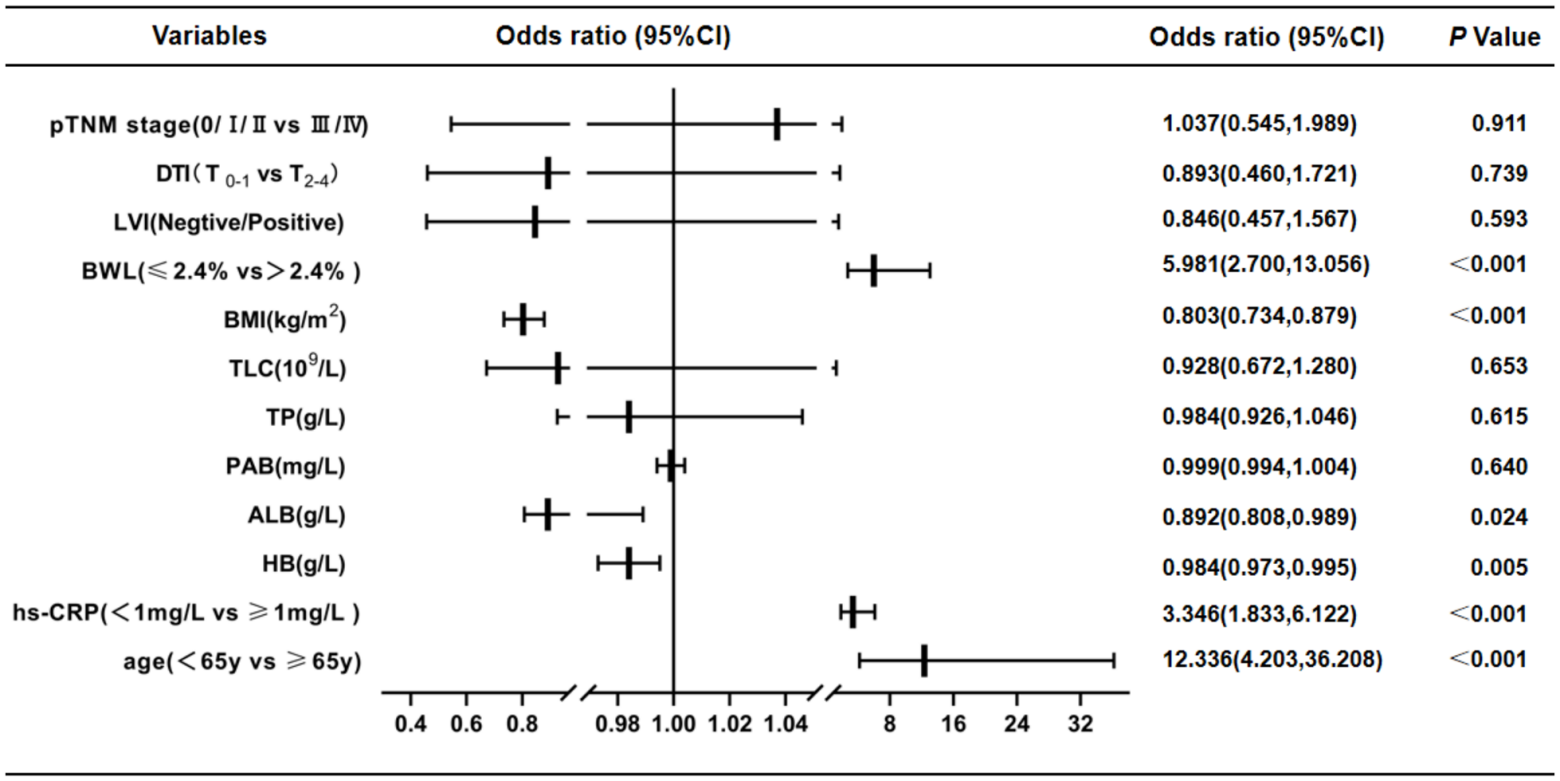
Results of multivariate logistic regression analysis of malnutrition in GC patients by PG-SGA(scores ≤ 1 point, grade A vs. ≥ 2 points, grade B + C). These findings presented that age, hs-CRP, HB, ALB, BMI and BWL were independent risk factors for malnutrition in GC. CI, confidence interval; DTI, depth of tumor invasion; LVI, lymph-vascular invasion; BWL, body weight loss; BMI, body mass index; TLC, total lymphocyte count; TP, total protein; PAB, prealbumin; ALB, albumin; HB, hemoglobin; hs-CRP, hypersensitive C-reactive protein.

According to PG-SGA nutrition assessment scale, GC patients were divided into two groups: non-malnutrition (scores ≤ 1 point, grade A) and suspected or moderate-to-severe malnutrition (scores ≥ 2 points, grade B + C). Univariate logistic regression analysis displayed that age (*P* < 0.001), hs-CRP (*P* < 0.001), HB (*P* < 0.001), TP (*P* < 0.001), ALB (*P* < 0.001), PAB (*P* < 0.001), TLC (*P* < 0.001), BMI (*P* < 0.001), BWL (*P* < 0.001), LVI (*P* = 0.034), DTI (*P* = 0.004) and pTNM stages (*P* = 0.041) were risk factors for malnutrition in GC patients (**Table 4**). Multivariate logistic regression analysis was performed on the factors of significant statistical differences in the above from univariate analysis, and the findings presented that age (OR = 12.336, 95% CI: 4.203-36.208; P < 0.001), hs-CRP (OR = 3.346, 95% CI: 1.833-6.122; *P* < 0.001), HB (OR = 0.984, 95% CI: 0.973-0.995; *P* = 0.005), ALB (OR = 0.892, 95% CI: 0.808-0.989; *P* = 0.024), BMI (OR = 0.803, 95% CI: 0.734-0.879; *P* < 0.001) and BWL (OR = 5.981, 95% CI: 2.700-13.056; *P* < 0.001) were independent risk factors for malnutrition in GC (**Figure 2**).

**Table 4:** Univariate logistic regression analysis of malnutrition in patients with GC by PG-SGA (grade A vs. grade B+C).

Variables	Odds ratios (95% CI)	*P* Value
Gender	1.299(0.825,2.046)	0.258
Age(<65 y vs. ≥65y)	13.074(4.734,36.096)	<0.001
hs-CRP(<1.00mg/L vs. ≥1.00mg/L)	3.536(2.214,5.654)	<0.001
HB	0.972(0.961,0.982)	<0.001
ALB	0.809(0.761,0.860)	<0.001
PAB	0.990(0.987,0.994)	<0.001
TP	0.892(0.857,0.928)	<0.001
TLC	0.577(0.442,0.753)	<0.001
BMI	0.847(0.794,0.904)	<0.001
BWL(≤2.4%/>2.4%)	6.922(3.292,14.555)	<0.001
Grade(Well/moderate vs. Poor/undifferentiated)	0.721(0.426,1.221)	0.223
TMD(<5cm/≥5cm)	1.602(0.986,2.604)	0.057
PNI(Negtive/Positive)	1.323(0.853,2.053)	0.211
LVI(Negtive/Positive)	1.646(1.039,2.608)	0.034
DTI(T_0-1_ vs. T_2-4_)	1.953(1.234,3.092)	0.004
LNM(Negtive/Positive)	1.513(0.981,2.334)	0.061
pTNM stages (0/I/II vs. III/IV)	1.565(1.018,2.406)	0.041

CI, confidence interval; hs-CRP, hypersensitive C-reactive protein; HB, hemoglobin; ALB, albumin; PAB, prealbumin; TP, total protein; TLC, total lymphocyte count; BMI, body mass index; BWL, body weight loss; TMD, tumor maximum diameter; PNI, peripheral nerve invasion; LVI, lymph-vascular invasion; DTI, depth of tumor invasion; LNM, lymph node metastasis.

## Discussion

GC is a malignant tumor with high morbidity and mortality. The pathological features and nutritional status of patients greatly influence GC progression. In China, most GC patients were found to be in an advanced stage with lymph node metastasis (23). In this study, 76.8% of patients had advanced GC while 65.9% of patients were having lymph node metastasis, respectively, which was mainly related to the failure of timely detection because of the unavailability of active regular gastroscopy. Furthermore, this study found that PNI and LVI were common in GC, with 40.0% and 38.9% incidence rates, respectively, similar to 35.9% and 34.3% reported rates by the other scholars (24, 25). GC, along with malnutrition, is also a common issue. A study in Japan showed that 60% of GC patients had malnutrition (5). This study reported more than 80% of suspected or moderate-to-severe malnutrition cases because most patients included had advanced GC.

The hs-CRP is a frequently used factor of inflammatory response. Previously, more attention was paid to its role in inflammation, but studies have found that hs-CRP is related to tumor occurrence, progression, and nutrition in recent years. Elevated serum hs-CRP could intensify the risk of developing cancer (19). A prospective clinical study on primary liver cancer reported that serum hs-CRP ≥ 1 mg/L was associated with the occurrence of primary liver cancer (26). Similarly, research on breast cancer has illustrated that high serum hc-CRP levels could increase the risk of breast cancer (27). In lung cancer studies, it was found that along with lung adenocarcinoma, the upsurge of circulating hs-CRP concentration was significantly correlated with many other histological types of lung cancer, such as large cell carcinoma, small cell carcinoma, squamous cell carcinoma, where the hs-CRP > 1 mg/L elevated the risk of death due to lung cancer (28, 29). In the study of colorectal cancer, hs-CRP > 5 mg/L before treatment was associated with LNM, PNI, LVI, low tumor grade, distant metastasis, and increased pTNM stage, but not with age and gender. The five-year survival rate of colorectal cancer patients with hs-CRP > 5 mg/L before treatment was significantly reduced. Multivariate analysis suggested that hs-CRP > 5 mg/L before treatment was an independent risk factor affecting the prognosis of colorectal cancer patients (30). In advanced GC, Woo et al. (31) reported that hs-CRP was related to age, DTI, LNM, LVI, PNI, pTNM stage, and tumor recurrence, but not histological type, tumor grade, and site. After radical resection, the hs-CRP > 3 mg/L was an independent risk factor for GC recurrence. This study derived similar results. Our findings elaborated that preoperative serum hs-CRP ≥ 1 mg/L was significantly correlated with age, TMD, PNI, LVI, DTI, LNM, and pTNM stages of GC patients, but not with gender and tumor grade. In brief, the pathological characteristics of variable hs-CRP levels might not be the same in different tumors, which warrants additional investigation.

Pro-inflammatory cytokines are associated with cancer-related malnutrition and cachexia (32). CRP or hs-CRP is a crucial inflammatory factor, and few studies have been conducted on serum CRP or hs-CRP in tumor nutrition. Yilmaz et al. (33) explained that high levels of CRP were significantly related to malnutrition in patients with hematological malignancies. Some scholars divided BMI into two groups (< 25 kg/m^2^ and ≥ 25 kg/m^2^) in advanced GC, but the results exhibited no significant correlation between hs-CRP and BMI (31). However, this research found that preoperative serum hs-CRP ≥ 1 mg/L was significantly associated with BMI, owing to the discrepancy between the hs-CRP threshold used in this study and those used by other researchers. Meanwhile, the hs-CRP level was also revealed to be significantly interrelated with BWL, HB, TP, ALB, PAB, and TLC in GC patients. It was worth mentioning that the results of this study showed that preoperative hs-CRP ≥ 1 mg/L was significantly linked with NRS2002 score ≥ 3 points (existing malnutrition risk) and PG-SGA score ≥ 2 points (suspected or moderate-to-severe malnutrition). Therefore, it was believed that preoperative serum hs-CRP level had good clinical value for evaluating the nutritional status of GC patients.

Based on the above results, the univariate logistic regression analysis was further conducted on the impact of nutritional risk screening (NRS2002 scale) and malnutrition assessment (PG-SGA scale) on GC patients. The factors with significant statistical differences in univariate analysis results were analyzed by multivariate logistic regression. Multivariate analysis results displayed that age ≥ 70 years, hs-CRP ≥ 1 mg/L, low ALB, low BMI, BWL > 2.4%, and TMD ≥ 5 cm were independent risk factors for existing nutritional risk in GC patients. Age ≥ 65 years, hs-CRP ≥ 1 mg/L, low ALB, low HB, low BMI, and BWL > 2.4% were independent risk factors for malnutrition in GC patients. Currently, age, BMI, and BWL are common scoring indexes for clinical nutrition evaluation (34, 35), in NRS2002 nutritional screening scale and PG-SGA nutritional assessment scale (10, 21), and ALB and HB are also frequently used biochemical indexes to evaluate malnutrition (36–38). This research also showed the clinical significance of these indexes in nutritional status in GC. In recent years, the malnutrition criteria that have been proposed by the Global Leadership Initiative on Malnutrition (GLIM) include three phenotypic criteria (involuntary weight loss, low BMI, and less muscle mass) and two etiological criteria (reduced feeding or digestive and absorption disorders, inflammation or disease burden), and inflammatory indicators including CRP, ALB and PAB (39). According to this study, preoperative serum hs-CRP plays an obvious role in the nutritional screening and evaluation of GC. It may have more clinical relevance due to its higher sensitivity than standard CRP detection. However, the precise mechanism of hs-CRP raising the risk of malnutrition in cancer patients is unknown. An animal cancer model demonstrated that pro-inflammatory cytokines and other factors could interfere with the interactions between metabolism-related organs (including skeletal muscle, adipose tissue, liver, and central nervous system), resulting in the destruction of metabolic dynamic balance, negative nitrogen balance, increasing skeletal muscle protein consumption, thereby exacerbating malnutrition (40). This could explain the negative correlation between high hs-CRP and malnutrition in tumor patients, although the specific mechanism needs to be researched further.

This study had some limitations. First, it was a retrospective study with a relatively small number of cases. In the future, we will carry out a prospective clinical study with a large sample size to enhance the reliability of the study. Second, because the patients in this study were not followed up on, the impact of postoperative hs-CRP changes on the long-term nutritional status of GC patients could not be assessed. Subsequent studies will be conducted to determine further the clinical value of hs-CRP in nutritional screening and evaluation of GC patients.

## Conclusion

To conclude our findings, preoperative serum hs-CRP levels aid in evaluating TMD, PNI, LVI, DTI, LNM, and pTNM stages to some extent. They may also be an effective indicator of nutritional risk screening and nutritional evaluation in GC patients.

## Data availability statement

The raw data supporting the conclusions of this article will be made available by the authors, without undue reservation.

## Ethics statement

The studies involving human participants were reviewed and approved by the ethics committee at the First Affiliated Hospital of Guangxi Medical University. The patients/participants provided their written informed consent to participate in this study.

## Author contributions

The authors' responsibilities were as follows–JQC designed the study. YTM, JLL, and YQ performed the statistical analysis. JQC, YTM, and JLL wrote the paper. JML, ZW, BPL, SYL, and LT collected and analyzed the data. All authors contributed to the article and approved the final version of manuscript.
